# Secondary Traumatic Stress, Mental State, and Work Ability in Nurses—Results of a Psychological Risk Assessment at a University Hospital

**DOI:** 10.3389/fpsyt.2020.00298

**Published:** 2020-04-27

**Authors:** Christian Bock, Ivo Heitland, Tanja Zimmermann, Lotta Winter, Kai G. Kahl

**Affiliations:** ^1^ Department of Occupational Safety, Hannover Medical School, Hannover, Germany; ^2^ Department of Psychiatry, Social Psychiatry and Psychotherapy, Hannover Medical School, Hannover, Germany; ^3^ Department of Psychosomatic Medicine and Psychotherapy, Hannover Medical School, Hannover, Germany

**Keywords:** secondary traumatic stress, nurse, work strain, depression, anxiety, workload

## Abstract

Psychological risk assessment is a legal obligation for companies and part of occupational safety and employment protection in Germany. However, data from psychological risk assessments in nursing staff are scarce, although this population is at increased risk for secondary traumatic stress by patient experienced trauma. Therefore, our study aimed at examining the frequency of reported secondary trauma events, secondary traumatic stress, and its possible consequences for psychological well-being and work ability in nurses. N = 320 nurses (n = 280 female) were assessed at a University Hospital in Germany as part of the psychological risk assessment. Secondary traumatic events, secondary traumatic stress, and symptoms of depression and anxiety were measured using self-report questionnaires (PHQ-2, GAD-2), and work ability was assessed using a modified version of the questionnaire for workplace analysis (KFZA). Of 320 nurses, 292 (91.2%) experienced secondary trauma, and 74 nurses (25.3%) reported secondary traumatic symptoms. Nurses with secondary traumatic symptoms reported higher depression (p < 0.001) and anxiety scores (p < 0.001) compared to nurses without secondary trauma experience, and to nurses with secondary trauma experience but without secondary traumatic stress (both p < 0.001). Further, nurses with secondary traumatic stress reported significantly reduced work ability, social support and control over work, and increased emotional strain and labor time. Nurses with secondary traumatic stress may be at increased risk of developing major depression and anxiety disorders, and particularly need support in overcoming secondary traumatic experiences. Psychological risk assessment is a useful tool to identify groups at risk, and pave the way to implement strategies to improve mental well-being and prevent work ability in high risk groups.

## Introduction

Work-related stress belongs to the most concerning issues in occupational health in industrialized countries (1). Stress is experienced by approximately 45% of European employees, and is seen as the second most important threat after musculoskeletal problems posed by the working environment (2). Studies supports the association between perceived stress, physical and mental health, being probably linked *via* stress axis activation (3).

The implementation of psychological risk assessments has been a legal obligation for German companies since 2013 (4). Although there are no regulations regarding the instruments used to assess psychological risk at work, it was proposed to use questionnaires and/or structured interviews to capture data from employees. This offers the opportunity to assess work-related psychological risk factors such as secondary traumatic stress and relate them to measures of psychological health and work ability.

Among hospital workers, patient care professionals are more vulnerable to develop work-related health problems compared to other professions (5). Among others, secondary traumatic stress (STS) is an occupational hazard for persons who provide direct patient care. STS is defined according to Figley (1995) as “the natural consequent behaviors and emotions resulting from knowledge about a traumatizing event experienced by a significant other. It is stress resulting from helping, or wanting to help a traumatized or suffering person” (6).

The experience of STS has been reported frequently in many caring professions including nursing. There are several published studies examining STS and closely related conditions in different nursing specialties such as emergency care (7–9), critical care (10), cancer/oncology (11, 12), internal medicine/heart and vascular nurses (13), primary care (14), labor and delivery (15), psychiatric care (16), and pediatric care (17, 18). These studies demonstrated that STS is a frequent condition across nursing specialties, with prevalence rates ranging between 35% and 60% (19).

Nurses who experienced STS were described to suffer emotionally as a result of shared traumas with their patients. They may have recurrent thoughts or distressing dreams, sleep disturbances, and even flashbacks of difficult patient experiences have been described. Furthermore, they may have difficulties performing their jobs because of psychological distress (8).

We here report data from a cohort of nurses working in a university hospital in Germany who underwent psychological risk assessment between 2016 and 2018. We aimed at examining the frequency of secondary traumatic experiences, STS, and their potential association with measures of psychological and work-related problems. Our primary hypothesis was that individuals who reported secondary traumatic stress have increased anxiety and depression scores.

## Methods

### Participants

This study was approved by the local ethic committee (Hannover Medical School). Psychological risk assessment was performed in 1,057 participants working in different professions and in different Departments at the Hannover Medical School. Participants were asked to fill in a questionnaire anonymously as a part of the psychological risk assessment process. Of the 1,057 participants 338 were nurses, of whom 320 questionnaires were complete.

### Measures

Secondary traumatic stress was assessed using a questionnaire with two questions which are part of the Freiburg PTSD Screening (20). Participants were asked whether they experienced traumatic events at work (yes/no), and whether they currently suffer from flashbacks regarding traumatic events at work (yes/no). These data were used to classify subjects based on STE/STS into three distinct groups. If no traumatic work event was reported, subjects were classified as the “no STE” group. If a traumatic event at work was reported without flashbacks, subjects were classified as experience of secondary traumatic event without secondary traumatic stress, the “STE without STS” group. If flashbacks were affirmed in the context of this traumatic experience, subjects were classified into the “STE with STS” group.

Symptoms of depression and anxiety were assessed using the two item Patient Health Questionnaire-2 (PHQ-2) (21) and the two item Generalized Anxiety Disorder scale (GAD-2) (22). Both scales pertain to symptom frequency during the last two weeks using a 4-point Likert scale (0-3) ranging from “Not at all” (0) to “Almost every day” (3). Scores for both questionnaire range from 0 to 6, with ≥3 being used as the optimal cutoff point for screening purposes. The PHQ-2 assessed the frequency of depressed mood and anhedonia (“Loss of interest” and “Dejection or hopelessness”). Sum scores ≥3 suggest a major depressive disorder (23). The GAD-2 assessed core anxiety symptoms, i.e. tension and uncontrollable worry (“Nervousness or tension” and were “Not being able to stop or control worries”). Scores ≥3 points suggest presence of an anxiety disorder (24).

Age was measured on a 5-point ordinal scale consisting of the items “up to 25 years”, “26–35 years”, “36–45 years”, “46–55 years”, “56 years or more”.

Work strain and work ability were assessed using a modified version of the questionnaire for workplace analysis (KFZA; Prumper, Hartmannsgruber and Frese (25). The final questionnaire consisted of 7 scales with two items each: Questions about workload (two items, α = 0.70), control over work (two items, α = 0.70), social support (two items, α = 0.72), workflow (two items, α = 0.70), feedback (two items, α = 0.64), work environment (two items, α = 0.60), and information/participation (two items, α = 0.70). Denominators ranged from “I strongly agree”, “I agree”, “Neither agree nor disagree”, to “I disagree” and “I strongly disagree”.

### Data Analysis

All statistical analyses were conducted using SPSS version 25. Descriptive analyses were performed for the whole group concerning age, gender, marital status, nursing specialty, secondary traumatic events and secondary traumatic stress, and occupational conditions.

Group differences concerning nominal variables were compared using Chi square tests. To examine the effects of STE/STS, MANOVA’s were performed using STE/STS as independent variable with the 3 levels “no STE”, “STE without STS” and “STE with STS”, and measures of psychological health (PHQ-2, GAD-2;MANOVA #1) as well as measures of work strain and work ability (MANOVA #2) as dependent variables. Finally, Bonferroni-corrected post-hoc tests were used for pairwise comparisons.

## Results

Eighty-eight percent of the sample were female, and most were in the age range between 26-35 years. One-hundred thirty (40.6%) were working in internal medicine, 78 (24.4%) in surgery, 63 (19.7%) in pediatric care, and 49 (15%) in psychiatric care (**Table 1**). Most nurses were partnered (207, 64.7 %) .

**Table 1:** Psychosocial and occupational data.

Factor	All nurses (N/%)
Female Gender (N/%)	280 (87.5%)
Age range (y)	
≤25	51 (15.9%)
26-35	111 (34.7%)
36-45	66 (20.6%)
46-55	67 (20.8%)
≥56	25 (7.8%)
Nursing specialty	
Surgical care	78 (24.4%)
Pediatric care	63 (19.7%)
Psychiatric care	49 (15.3%)
Internal medicine	130 (40.6%)
Secondary traumatic event	292/320 (91.2%)
Secondary traumatic stress	74/292 (25.3%)
Marital status	
Singled	87 (27.2%)
Married/partnership	207 (64.7%)
Divorced	22 (6.9%)
Widowed	4 (1.3%)
Occupational condition	
Support by peers	251 (78.5%)
Support by supervisor	172 (53.8%)
Shift work	268 (83.8%)
Overtime hours	160 (50%)
Security of employment	298 (93.1%)
Opportunity of advancement	71 (22.2%)

Secondary traumatic events were reported by 292 nurses (91.2%), of whom 74 (25.3%) reported that these events lead to continuous rumination and/or flashbacks (**Table 1**).

A MANOVA with group (no STE; STE without STS; STE with STS) as independent variable, and psychological health as dependent variables (PHQ-2, GAD) showed a significant multivariate effect [Wilks Lambda = 0.88, F(4, 632) = 10.5, p < 0.001, partial *η* ^2^ = 0.06]. Corresponding univariate tests showed significant group effects for both symptoms of anxiety (GAD-2 sum score) [F(2,317) = 16.0, p < 0.001, partial *η* ^2^ = 0.09] and symptoms of depression (PHQ-2 sum score) [F(2,317) = 20.3, p < 0.001, partial *η* ^2^ = 0.11].

**Figure 1:**
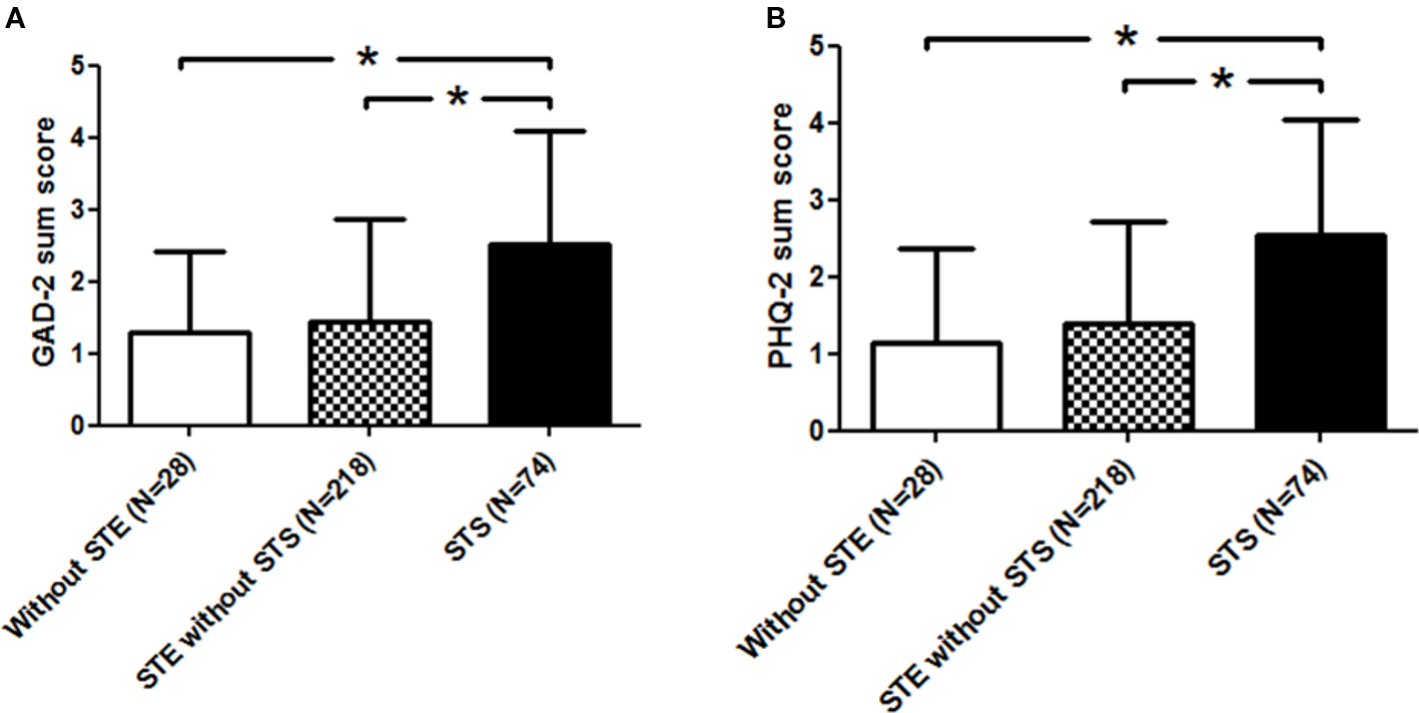
**(A)** Increased symptoms of anxiety symptoms in nurses who reported secondary traumatic stress (STS) compared to both other groups. *Indicates a significance level <0.001. **(B)** Increased symptoms of depression in nurses who reported secondary traumatic stress (STS) compared to both other groups. *Indicates a signifcance level <0.001.

Post-hoc analyses for symptoms of anxiety revealed higher GAD-2 sum score in nurses who experienced secondary traumatic stress compared to nurses without experience of a secondary traumatic event (p < 0.001), and compared to nurses who had experienced secondary traumatic event with developing secondary traumatic stress (p < 0.001) (**Figure 1A**).

Post-hoc analyses for symptoms of depression revealed higher PHQ-2 sum score in nurses who experienced secondary traumatic stress compared to nurses without experience of a secondary traumatic event (p < 0.001), and compared to nurses who had experienced secondary traumatic event with developing secondary traumatic stress (p < 0.001) (**Figure 1B**).

Further analyses were made to identify possible group associations with regard to measures of work strain and work ability. There was a significant multivariate effect of group on work strain and work ability [Wilks Lambda = 0.87, F(14, 596) = 2.7, p = 0.001, partial *η* ^2^ =.06]. Corresponding univariate tests showed significant group effects for workload [F(2,304) = 8.3, p < 0.001, partial *η* ^2^ =.05], social support [F(2,304) = 4.6, p = 0.011, partial *η* ^2^ =.03], workflow [F(2,304) = 10.2, p < 0.001, partial *η* ^2^ =.06] and participation [F(2,304) = 7.1, p = 0.001, partial *η* ^2^ =.04], but no significant effects for control over work and planning [F(2,304) = 2.9, p = 0.057], work environment [F(2,304) = 1.7, p = 0.18] and feedback [F(2,304) = 1.3, p = 0.27]. The corresponding descriptive statistics for all work-related data are shown in **Table 2**.

**Table 2:** Descriptive statistics for age, gender, and nursing specialty, work strain and work ability dependent on secondary traumatic experiences (no STE, STE without STS, STE with STS).

	No STE (N=28)	STE without STS (N=218)	STE with STS (N=74)
Gender			
Female	26 (9.3%)	186 (66.4%)	68 (24.3%)
Male	2 (5.0%)	32 (80%)	6 (15%)
Age range			
≤25y	8 (15.7%)	32 (62.7%)	11 (21.6%)
26-35y	9 (8.1%)	71 (64.0%)	31 (27.9%)
36-45y	5 (7.6%)	48 (72.7%)	13 (19.7%)
46-55y	4 (6.0%)	48 (71.6%)	15 (22.4%)
≥55y	2 (8.0%)	19 (76.0%)	4 (16%)
Nursing specialty			
Surgical care	7 (9.0%)	54 (69.2%)	17 (21.8%)
Internal medicine	14 (10.8%)	84 (64.6%)	32 (24.6%)
Pediatric care	3 (4.8%)	44 (69.8%)	16 (25.4%)
Psychiatric care	4 (8.2%)	36 (73.5%)	9 (18.4%)
Workload			
Being under pressure	3.6 ± 0.8	3.8 ± 0.8	4.1 ± 0.7 ^a,b^
Having too much work	3.2 ± 0.9	3.3 ± 0.8	3.7 ± 0.8 ^a,b^
Control over work			
Influence on amount of work	3.5 ± 0.7	3.6 ± 0.7	3.7 ± 0.8
Plan work independently	3.7 ± 0.9	3.3 ± 0.9 ^a^	3.0 ± 0.8 ^a,b^
Social support			
Support by colleagues	4.1 ± 0.6	4.0 ± 0.8	3.7 ± 0.7 ^a,b^
Support by supervisor	3.5 ± 1.1	3.5 ± 1.1	3.1 ± 1.1 ^a,b^
Workflow			
Information or equipment not available	2.4 ± 0.8	2.5 ± 0.8	3.0 ± 1.1 ^a,b^
Work often interrupted	3.6 ± 1.0	4.0 ± 0.8 ^a^	4.3 ± 0.7 ^a,b^
Feedback			
Appropriate feedback by colleagues	2.7 ± 0.9	2.8 ± 1.0	3.1 ± 1.1
Appropriate feedback by supervisors	2.7 ± 0.9	3.0 ± 1.2	3.2 ± 1.1
Work environment			
Stressful work environment	3.5 ± 1.3	3.6 ± 1.2	3.9 ± 1.2
Equipment inadequate	3.2 ± 1.2	3.6 ± 1.1	3.6 ± 1.2
Information and participation			
Always kept up-to-date	3.5 ± 0.8	3.4 ± 0.8	2.9 ± 1.1 ^a,b^
Superiors consider employees ideas	2.3 ± 1.0	1.6 ± 0.7 ^a^	1.6 ± 0.7 ^a,b^

^a^Means a group difference (P < 0.05) between “STE with STS” and “no STE”. ^b^Means a group difference (P < 0.05) between “STE with STS” and “STE without STS”. STS, secondary traumatic stress.

Post-hoc analyses revealed that nurses from the “STE with STS” group reported significantly more frequent feelings of “being under pressure” and “having too much work” (workload scale) compared to nurses from the “no STE” (p = 0.005) and nurses from the “STE without STS” groups (p < 0.001, see **Table 2** for descriptive statistics). Furthermore, nurses from the “STE with STS” group reported less support by colleagues and supervisors (support scale) than nurses from the “STE without STS” group (p = 0.010). In addition, nurses from the “STE with STS” group reported being less being informed/well equipped and more often interrupted (workflow scale) than nurses from the “STE without STS” group and the “no STE” group (both p-values < 0.001). Finally, “STE with STS” nurses reported being less kept up-to-date with information and ideas/less being considered by superiors (participation scale) than “STE without STS” (p = 0.002) and “no STE” (p = 0.015) nurses. There were no group effects with regard to age, gender and nursing specialty (all p-values n.s). See **Table 2** for an overview of descriptive statistics regarding all post-hoc tests and distributions.

## Discussion

This study assessed the frequency of secondary traumatic experiences, secondary traumatic stress, mental health problems and work ability in 320 nurses from a university hospital. We demonstrate high rates (> 90%) of self-reported secondary traumatic experiences in nurses of different professions. Those who developed STS (25.3%) displayed more symptoms of depression and anxiety, experienced higher job strain, and lower work ability.

The psychological and work-related consequences of secondary traumatic stress in nurses have seldom been studied. Reports so far demonstrated relatively high levels of STS in nurses (19), described risk factors (26) and emotional consequences such as compassion fatigue and burnout (27, 28). Our study expands these studies by showing high amounts of secondary traumatic experiences in nurses from different nursing specialties, demonstrating that those who are burdened by secondary traumatic stress report higher levels of anxiety and depression symptoms, and demonstrating consequences in the workplace. In particular, we found that those afflicted by STS reported higher job strain, less social support by colleagues and supervisors, and less participation.

The combination of STS with psychological burden and less social support by peers may pave the way to further problems such as the development of anxiety disorders, major depression (29, 30), and absenteeism from work (31). Predictors of absenteeism in nurses have been reviewed by Davey and colleagues, and high job strain, low peer or supervisor support, low work control, and high role overload were identified as important factors (31). Several of the aforementioned factors were also found in our sample of nurses with STS.

Other factors leading to the experience of job strain have been studied intensively. Job characteristics, such as high work demands in combination with limited autonomy and support were shown to contribute to an overall experience of job strain. (32, 33). Further, personality traits may play an important role in perceiving job demands as stressful. E.g., type D personality has been proposed to consist of distress-prone characteristics, i.e. inhibited interpersonal interactions and a predisposition towards negative affectivity (34). Type D personality traits have been associated with higher levels of depressive symptoms, overall mental stress and health, higher rates of disability and higher rates of sick leave (35–37). In a recent study by Duan-Porter and colleagues, personality traits of negative affectivity accounted for 36% of between-individual variation in depressive symptoms over 12 months, and job characteristics and coping explained an additional 5% and 8% (38).

Individual coping strategies may also influence perceived job strain and the development of depressive symptoms. Individuals who are at greater risk for depression have more avoidant coping strategies, while individuals who deal proactively with stressors and use social support seem to be less prone to depression (39–41).

Since personality traits and coping strategies were not assessed in our study, we cannot rule out whether personality traits of nurses with difficulties to cope with patients’ situations may influence the perception of secondary traumatic event and secondary traumatic stress. Further, we cannot comment on the question whether workplace-related stress may add or multiply the risk of secondary traumatic events.

### Implications for Practice

Based on our findings we recommend including measures of secondary traumatic events and secondary traumatic stress symptoms in the psychological risk assessment process for nurses and for employees of other professions who directly care for patients in hospitals. Measures of individual coping strategies and personality factors may also be integrated.

An open question applies to whether a 2-question solution like in our study might be sufficient, or whether more detailed questionnaires are advantageous. E.g., a more detailed analyses of secondary traumatic stress symptoms can be achieved using the Secondary Traumatic Stress Scale (42), that corresponds to the symptoms of post-traumatic stress disorder listed in the Diagnostic and Statistical Manual of Mental Disorders (43). The STSS is composed of three subscales, namely intrusion, avoidance, and arousal. The instrument is designed to assess only secondary traumatic stress, and has achieved high levels of internal consistency reliability in published studies (19). However, one has to take in mind that data driven from a psychological risk assessment are part of occupational health and safety procedures. These assessments typically do not integrate sophisticated measurements as this is the case in clinical studies.

Second, based on our results and on other existing studies, comprehensive offers for nurses are recommended to provide continuous psychological support. Nurses need to be educated about their vulnerability when working with patients, about signs and symptoms of secondary traumatic stress, risk factors, and STS-related coping behaviors such as avoidant and numbing responses (44). Further, continuing education may be offered whereby nurses learn about ways how to prevent STS symptoms, and increase resilience to STS.

Several factors have been described that may enhance or prevent the occurrence of STS symptoms. According to Ratrout and Hamdan-Mansour, personal and organizational factors have to be considered to understand the process of developing STS symptoms (8). Personal factors include age (26), gender and years of working experience (7), educational level (45), trauma training, social support and personal trauma history (26) coping strategies (40) and personality factors (46). Organizational factors include trauma case load (26), peer and organizational support (47), and clinical supervision (48).

Rourke (2007) identified three areas of strategies (personal, professional, organizational) for preventing or ameliorating STS (49). On the personal level, strategies such as a healthy lifestyle (such as getting enough sleep, regular exercise, healthy diet), enjoying leisure time activities, managing a good work-family balance, permitting adequate time for grieving lose patients, and eventually using psychotherapy for those care providers who have strong emotional responses in the context of STS were recommended (49).

On the professional level, suggested strategies comprised peer consultation, setting boundaries, and/or meeting regularly with respectful professionals; and on the organizational level, strategies such as establishing a respectful and encouraging atmosphere and creating a support team were recommended (49).

These offers may include external and/or internal supervision, group intervision, and continuous quality management. Hospitals are recommended to offer opportunities for crisis intervention to nurses (and other employees) after a secondary work-related traumatic event. Our results demonstrate the effect of (perceived or real) lack of social support by colleagues and superiors. Therefore, a positive working atmosphere, offers to promote collegiality, an appreciative and constructive feedback culture should be mandatory.

### Limitations

The nature of the traumatic stressors, and psychological factors such as subjective interpretation of the traumatic stressor were not assessed in our study. Further, due to the cross-sectional design of the study we can only report associations, and cannot infer causality. The data presented here belong to the psychological risk assessment, which is a legal obligation for companies in Germany. The questionnaire used here had to be approved by the Employee Committee. Some interesting facets of our results, such as personality traits, coping mechanisms, and ability for the job, were not approved.

## Conclusion

We found high rates of secondary traumatic experiences in nurses of different professions. Those who developed secondary traumatic stress displayed more symptoms of depression and anxiety, higher job strain and lower work ability. We conclude that secondary traumatization and secondary traumatic stress symptoms should not be regarded as a lack of resilience, but as an occupational hazard of the nurses’ working environment. The issuing of measures to reduce secondary traumatic stress symptoms is an opportunity for hospitals to promote mental health, work ability and commitment to the company by their employed nurses.

## Data Availability Statement

The raw data supporting the conclusions of this article will be made available by the authors, without undue reservation, to any qualified researcher.

## Ethics Statement

The studies involving human participants were reviewed and approved by the Local Ethics Committee of the Hannover Medical School, Hannover, Germany. Written informed consent for participation was not required for this study in accordance with the national legislation and the institutional requirements.

## Author Contributions

CB, KK, and TZ designed the study. CB collected the data. CB and KK conducted the analyses. TZ supervised the method and statistical analyses. KK and LW wrote the manuscript. IH revised the statistical section. All authors made substantial contributions to the conception or design of the work, or the acquisition, or interpretation of data for the work; for drafting the work or revising it; approved the final version to be accountable for all aspects of the work.

## Conflict of Interest

KK has received speaker honoraria by Servier, EliLilly, Berlin Chemie, and Janssen-Cilag.

The remaining authors declare that the research was conducted in the absence of any commercial or financial relationships that could be construed as a potential conflict of interest.
